# End-tidal CO_2_ responses and ventilatory correlates during voluntary hyperventilation: a repeated-measures pilot study

**DOI:** 10.3389/fphys.2026.1890190

**Published:** 2026-09-30

**Authors:** Liping Wang, Lihao Xue, Fenglin Jia, Huibian Zhang, Lihua Yu, GuiYou Wang, Zhao Gu

**Affiliations:** Department of Research, People’s Liberation Army Air Force Special Medical Center, Beijing, China

**Keywords:** end-tidal CO_2_, linear mixed model, paced voluntary hyperventilation, repeated-measures, respiratory physiology, ventilatory correlates

## Abstract

**Background:**

Controlled voluntary hyperventilation is widely used to study the ventilatory control of end-tidal CO_2_ (PetCO_2_), but the breath-by-breath determinants of PetCO_2_ during an externally paced maneuver remain incompletely characterized. This repeated-measures pilot study aimed to describe PetCO_2_ changes and to quantify associations with ventilatory timing and expired gas output during paced voluntary hyperventilation.

**Methods:**

Thirty healthy males (age range 19.2–39.8) performed a metronome-paced voluntary hyperventilation maneuver at 30 breaths/min intended to last up to 3 min. Breath-by-breath data were recorded with a COSMED K5 (COSMED, Rome, Italy). After excluding breaths with implausible respiratory frequency, 2,298 breaths from 30 participants were analyzed with a linear mixed model (LMM) using a cubic B-spline for elapsed time, participant-specific random intercepts and random slopes, and fixed effects of minute ventilation (VE), expired CO_2_ output (VCO_2_), and expiratory time (Te). A generalized estimating equation (GEE) with AR(1) working correlation was a sensitivity analysis, and participant-level cluster-bootstrap CIs provided the primary inferential intervals.

**Results:**

Participant-level mean PetCO_2_ during the maneuver was 17.1 ± 1.8 mmHg (range 13.3–21.2 mmHg) and declined over time. In the primary LMM, VE β = −0.144, VCO_2_ β = 0.009, and Te β = 4.18. Because residual diagnostics showed non-normality, heteroscedasticity, and strong serial autocorrelation (GEE AR(1) alpha approximately 0.79 for the spline model and 0.86 for the linear-time model), cluster-bootstrap CIs are reported as the primary inferential intervals: VE −0.174 to −0.110, VCO2 0.005 to 0.012, Te 2.330 to 6.164, and a predicted PetCO2 change from 0 to 180 s of −8.58 mmHg (95% CI −10.30 to −6.92 mmHg). Marginal R^2^ = 0.775 and conditional R^2^ = 0.828; leave-one-participant-out cross-validation yielded R^2^ = 0.737 and RMSE = 2.52 mmHg.

**Conclusions:**

PetCO_2_ during paced hyperventilation was associated with elapsed time, VE, VCO_2_, and Te. Because VE and VCO_2_ are algebraically coupled, and VCO_2_ and PetCO_2_ also share information from the expired-CO_2_ signal, they should not be interpreted as independent physiological determinants; the principal contribution is the within-participant expiratory-time association. The findings are exploratory and require confirmation in clinical populations.

## 1 Introduction

Voluntary hyperventilation is a standard experimental maneuver in which ventilation is deliberately increased above metabolic demand, producing a fall in arterial and end-tidal CO_2_ tension (Lum, 1975; Gardner, 1996; Havenith et al., 2025; Yılmaz et al., 2025). It is used clinically to provoke symptoms in suspected hyperventilation syndrome and to study ventilatory control and cerebrovascular reactivity (Tiotiu et al., 2021; Pauwen et al., 2022; Brinkman et al., 2023). In healthy individuals, end-tidal CO_2_ (PetCO_2_) is commonly used as a non-invasive surrogate for arterial CO_2_ tension under stable breathing conditions, although the validity of this surrogate during rapid breathing depends on the attainment of a clear alveolar plateau and adequate instrument response time (Bhavani-Shankar et al., 1992; Bhavani-Shankar et al., 1995; Jaffe, 1996; Selki et al., 2024; Altarriba et al., 2025).

Previous work has described the average magnitude of PetCO_2_ decline during induced hyperventilation, but the breath-by-breath correlates of PetCO_2_ within and between individuals have received less attention. Minute ventilation (VE), expired CO_2_ output (VCO_2_), and expiratory timing are physiologically plausible determinants: higher VE removes more CO_2_ from the lung and body stores, whereas a longer expiratory time may allow the gas analyzer to sample later alveolar gas and thus record a higher end-tidal value. These variables are mathematically coupled (VE = respiratory frequency × tidal volume) and their effects are nested within individuals, so standard ordinary regression treating each breath as independent is inappropriate (Hurlbert, 1984; Gelman and Hill, 2007; Baayen et al., 2008; Lazic, 2010; Meteyard and Davies, 2020; Visalli et al., 2024).

The primary aim of this repeated-measures pilot study was to quantify the conditional associations between PetCO_2_ and ventilatory variables during a metronome-paced voluntary hyperventilation maneuver at 30 breaths/min intended to last 3 min in healthy young men, while separately describing within- and between-participant sources of variation in a secondary decomposition analysis. We hypothesized that PetCO_2_ would be negatively associated with VE and elapsed time, and positively associated with VCO_2_ and expiratory time. Because the maneuver produces a rapid early decline followed by a slower phase, we modeled elapsed time with a cubic B-spline rather than assuming a single linear slope.

## 2 Materials and methods

### 2.1 Participants

Thirty healthy adult male staff members were recruited from the People’s Liberation Army Air Force Special Medical Center between June and August 2025. Exclusion criteria were: (1) history of respiratory disease; (2) history of cardiovascular disease; (3) anxiety disorder or panic attacks; (4) current smoking; (5) medications affecting breathing; and (6) inability to perform voluntary hyperventilation. The study was approved by the Institutional Review Board at the People’s Liberation Army Air Force Special Medical Center (Approval No. 2025-54-PJ01) and all participants provided written informed consent.

The sample size of 30 participants was chosen based on convenience for this exploratory pilot sample. No formal power calculation was performed before data collection because the primary goal was estimation of associations and their uncertainty rather than definitive hypothesis testing. A small set of physiologically coherent fixed effects was selected on physiological grounds for the revised analysis, and all estimates are reported with 95% confidence intervals and sensitivity analyses.

### 2.2 Experimental protocol

The study protocol and data-processing pipeline are illustrated in **Figure 1**. Participants rested seated for 10 min in a temperature-controlled room (22–24 °C, 40%–60% relative humidity). They then performed a voluntary hyperventilation maneuver intended to last up to 3 min, paced at 30 breaths/min with a metronome set to 60 beats/min (one beat for inspiration, one for expiration), a method frequently used to standardize rapid voluntary breathing in research settings (Tabor et al., 2022). Verbal encouragement was given to maintain the target pattern. The test was terminated early if participants experienced severe dizziness, chest pain, or requested to stop. Continuous pulse oximetry and intermittent blood pressure measurements were performed, and all participants were observed during a recovery period until symptoms resolved. Participants were given a brief demonstration of the metronome-paced breathing before the maneuver.

**Figure 1:**
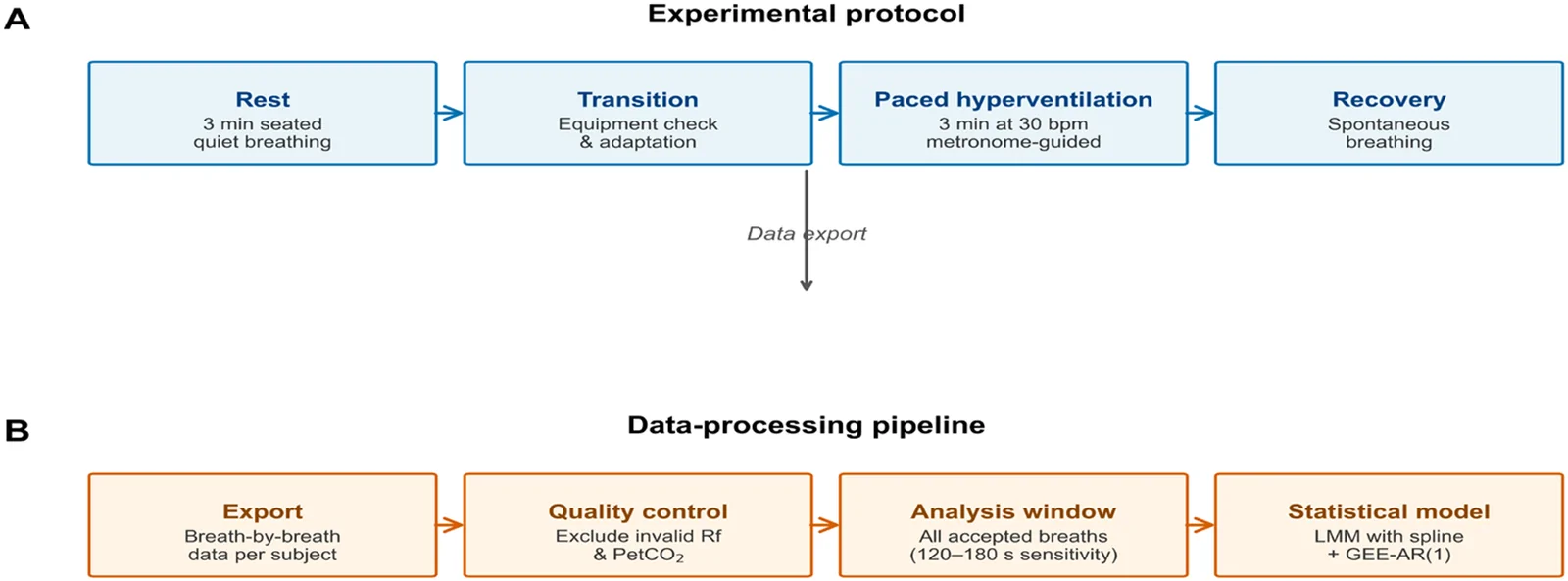
Study protocol and data-processing pipeline. **(A)** Experimental protocol. **(B)** Data-processing pipeline. LMM, linear mixed model; GEE, generalized estimating equation.

### 2.3 Measurements

A portable COSMED K5 breath-by-breath metabolic system (COSMED K5, Rome, Italy) was used. The flow sensor and gas analyzers were calibrated before each test according to the manufacturer protocol using a 3-L syringe for flow calibration and a known gas mixture (16% O_2_ and 4% CO_2_) for gas calibration (Kabbadj et al., 2024; Veneman et al., 2024). The following variables were recorded for each breath: PetCO_2_ (mmHg), respiratory frequency (Rf, breaths/min), tidal volume (VT, L), minute ventilation (VE, L/min), oxygen uptake (VO_2_, mL/min), expired CO_2_ output (VCO_2_, mL/min), inspiratory time (Ti, s), expiratory time (Te, s), and total breath duration (Ttot, s). VCO_2_ was calculated by the K5 software as the product of expired ventilation and expired CO_2_ fraction, after BTPS/STPD corrections. Ventilatory equivalents were computed as VE/VO_2_ and VE/VCO_2_. Volumes were corrected to BTPS and gas-exchange variables to STPD using the manufacturer software. Mask fit was checked before each test and participants were instructed to maintain a tight seal. We did not have an independent leak-detection algorithm; breaths with physiologically implausible respiratory frequency were excluded as described below. The K5 software version used was 2.4.2, and the mask model employed was COSMED S. The mean device-reported gas-transport delay—the time between exhaled CO_2_ leaving the mask and its detection by the CO_2_ analyzer, as estimated by the K5 software during its standard delay-calibration procedure—was 740 ms (range, 150–2,000 ms). This quantity is a gas-transport (sampling-line) delay, not the analyzer rise time; the K5 software applies this delay to align the flow and gas-concentration signals before computing breath-by-breath PetCO_2_ and VCO_2_. For each participant, time zero was defined as the timestamp of the first recorded breath of the maneuver, and elapsed time was computed relative to this point. The sampling-line length, mask dead space, and the breath-detection algorithm were not explicitly provided by the manufacturer and are therefore not available in this report.

### 2.4 Data quality control and analysis window

Because some participants deviated from the paced breathing target and the automated breath-detection algorithm produced physiologically implausible instantaneous respiratory frequencies, breath observations were excluded if Rf was <5 or >60 breaths/min or if PetCO_2_ was missing/invalid. This resulted in the exclusion of 446 of 2,744 raw breath observations (16.3%): 445 breaths for Rf outside the plausible range and one breath for an invalid PetCO_2_ value, leaving 2,298 breaths for analysis. All available accepted breaths within the intended 3-minute paced maneuver were analyzed; a sensitivity analysis restricted to the 120–180-s analysis window (observations occurring between 120 and 180 s among participants who reached ≥120 s) is also reported. The number of raw and accepted breaths per participant, the effective respiratory frequency, and the percentage of accepted breaths within the target 28–32 breaths/min window are summarized in the online supplementary data audit table.

Three complementary quantities were derived, and they are affected differently by breath-detection and exclusion artifacts. (1) Accepted observations per recorded minute, calculated as 60 × n_accepted/recorded_duration (seconds), is the number of accepted breath-by-breath observations per recorded minute. It is not the participant’s actual respiratory frequency, because excluded observations do not represent periods without breathing: they include detected breaths or artifacts rejected because the K5-reported Rf fell outside the acceptance range. Participant S05 illustrates this distinction: 172 breaths were detected during 180 s (effective Rf 34.1 breaths/min), but only 26 were accepted, yielding 8.7 accepted observations per recorded minute. (2) Effective respiratory frequency is the mean of the K5-reported instantaneous Rf across accepted breaths. (3) Timestamp-derived respiratory frequency is the mean of 60/Δt across consecutive accepted breaths; because excluded intervening observations can make an accepted-to-accepted interval span several actual breathing cycles, this metric underestimates the true rate when exclusions are frequent and cannot be regarded as an independent validation of the K5-reported Rf without this qualification. The percentage of breaths within the 28–32 breaths/min target window is the percentage among accepted breaths, not among all breathing cycles during the maneuver. Because accepted observations per recorded minute decreases as the exclusion rate rises, it reflects analytic data yield rather than breathing frequency, and we therefore do not use it as a measure of breathing frequency or as the primary measure of protocol adherence.

### 2.5 Statistical analysis

Analyses were performed in Python 3.12 using pandas 2.2.2, statsmodels 0.14.2, scikit-learn 1.5.1, and numpy 1.26.4. Descriptive statistics are presented as mean ± standard deviation (SD) or median and range, as appropriate. Because repeated breaths are nested within participants, the primary analysis was a linear mixed model (LMM) with a cubic B-spline for elapsed time (s), participant-specific random intercepts, and participant-specific random slopes for time (time_min), an approach that appropriately handles hierarchical data, avoids pseudoreplication, and does not assume a single linear time trend (Hurlbert, 1984; Gelman and Hill, 2007; Baayen et al., 2008; Lazic, 2010; Meteyard and Davies, 2020; Visalli et al., 2024). Elapsed time was represented by a cubic B-spline basis with 3 df and no interior knots; the model included a separate intercept, and the boundary knots were the minimum and maximum observed elapsed times. The exact Patsy formula for the fixed effects was PetCO_2_ ~ bs(time_sec, df = 3, degree = 3) + VE + VCO_2_ + Te (random-effect formula: ~time_min). The fixed-effects structure was selected on physiological grounds for the revised analysis: minute ventilation (VE), expired CO_2_ output (VCO_2_), and expiratory time (Te). Models were fitted by maximum likelihood (ML) using statsmodels 0.14.2. Marginal and conditional R² were computed following Nakagawa and Schielzeth, and the observation-averaged variance-partition coefficient was used because the random slope for time makes between-participant variance non-constant.

The marginal R^2^, conditional R^2^, and variance-partition coefficient (VPC) were computed from the estimated variance components to quantify clustering and explained variance. Because the model includes participant-specific random slopes for time, the between-participant variance is not constant; we therefore report an observation-averaged VPC rather than a single intraclass correlation coefficient (ICC). Random-effect variances were extracted from the covariance matrix of the random effects (cov_re) and the residual variance from the model scale; we did not report p-values or Wald confidence intervals for variance parameters because their sampling distributions are strongly skewed. A within/between-participant decomposition model was fitted by centering each predictor around its participant mean and including both the participant mean and the within-participant deviation as separate fixed effects. The decomposition used the same cubic B-spline for elapsed time as the primary model, with the exact fixed-effects specification PetCO_2_ ~ bs(time_sec, df = 3, degree = 3) + VE_m + VE_cw + VCO_2__m + VCO_2__cw + Te_m + Te_cw (subscript m denotes the participant mean; subscript cw denotes the within-participant deviation from the participant mean), together with participant-specific random intercepts and random slopes for time (time_min), fitted by ML. Repeated-measures correlations were computed for exploratory purposes using person-mean centering; 95% confidence intervals for both within- and between-participant correlations were obtained by a participant-level cluster bootstrap (2,000 resamples of participants, percentile intervals), because breaths within a participant are serially correlated and participants contribute unequal numbers of breaths.

Because residuals from adjacent breaths may be correlated, we also fitted a GEE with an AR(1) working correlation, using the same cubic B-spline time specification as the primary LMM (**Supplementary Table S6**). Observations were ordered by accepted-breath sequence within each participant, and AR(1) distance was based on this sequence; excluded breaths were removed and irregular intervals were handled by treating consecutive accepted breaths as adjacent. A linear-time GEE was retained as an alternative model for comparison, not as a direct sensitivity analysis of correlation structure alone. During bootstrap resampling, the spline basis was evaluated using the design information from the full dataset so that the boundary knots and spline construction remained identical across resamples. Participant-level cluster-bootstrap confidence intervals (2,000 resamples of participants) were computed for the primary LMM fixed effects, the overall time effect (predicted change in PetCO_2_ from 0 to 180 s), the within/between-participant decomposition coefficients, the 120–180-s late-window model, and the protocol-adherence sensitivity analyses. All bootstrap intervals are percentile intervals (the 2.5th and 97.5th percentiles of the bootstrap distribution). Within each resample, participants drawn more than once were assigned new unique cluster identifiers so that every copy carried independent random effects. All 2,000 fits converged successfully for every bootstrapped model; no fit failed, and no fallback to a simpler random-effects structure was required. These robust intervals are reported as the primary inferential intervals because residual diagnostics showed non-normality and heteroscedasticity and because the GEE indicated strong residual autocorrelation; model-based Wald results are retained as supplementary comparisons (**Supplementary Table S4**).

Model predictive performance was assessed using leave-one-participant-out cross-validation (LOPO-CV), in which the model was trained on all but one participant and predictions for the left-out participant were obtained from the fixed effects only. The root mean square error (RMSE) and coefficient of determination (R^2^) were reported. Two-tailed p <0.05 was considered significant.

## 3 Results

### 3.1 Participant characteristics and protocol adherence

All 30 participants performed the paced hyperventilation protocol without serious adverse events; recorded durations ranged from 85 to 184 s; the protocol permitted early termination for tolerability, although symptom-specific reasons for stopping were not systematically recorded. The cohort characteristics are shown in **Table 1**. After data quality control, the mean effective respiratory frequency was 33.1 ± 6.1 breaths/min. Only 54.6% ± 28.8% of accepted breaths fell within the 28–32 breaths/min target window (**Figure 2**). Twelve participants spent less than half of the maneuver within the target window, and three participants spent less than 10% within the target window, indicating heterogeneous adherence to the metronome pace despite verbal encouragement. The number of accepted breaths per participant ranged from 26 to 97 (mean 76.6; **Supplementary Table S1**). Raw breath counts ranged from 40 to 186; exclusion rates varied markedly, with three participants having >30% of raw breaths excluded (S05: 146/172, 84.9%; S26: 70/163, 42.9%; S28: 110/186, 59.1%).

**Table 1:** Participant characteristics (n = 30).

Characteristic	Mean ± SD	Range
Sex, n male/female	30/0	–
Age (years)	26.6 ± 6.9	19.2–39.8
Height (cm)	174 ± 5	168–192
Weight (kg)	73.7 ± 6.0	60.0–87.5
Body mass index (kg/m²)	24.2 ± 2.0	20.3–28.0

Height and body-mass-index data were missing for one participant (S15); n = 29 for these two characteristics.

**Figure 2:**
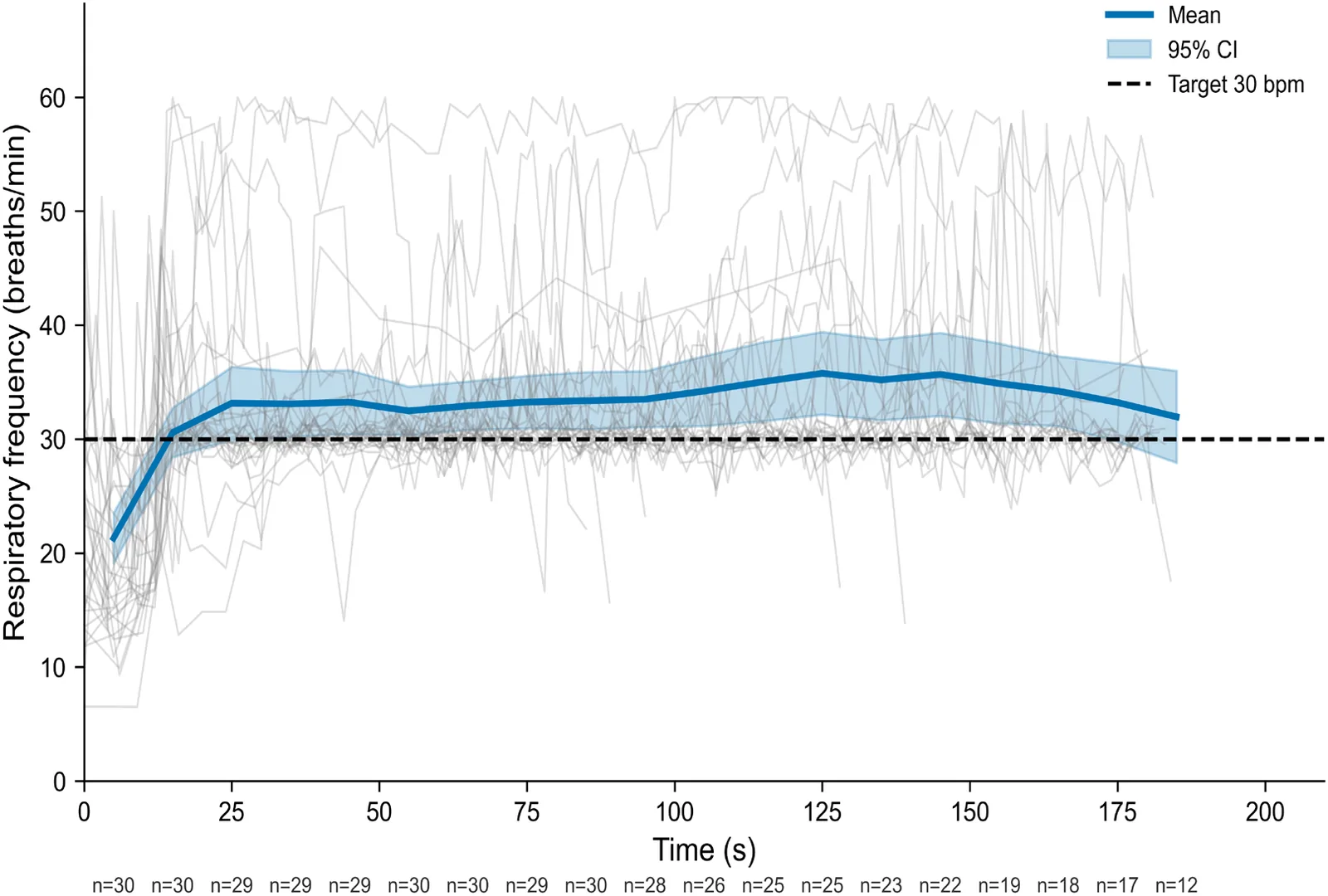
Breath-by-breath respiratory frequency during the paced hyperventilation maneuver. Each point represents one accepted breath; breaths excluded for physiologically implausible respiratory frequency are omitted. The solid blue line and shaded band show the mean and 95% CI within 10-s time bins. Because breaths are clustered within participants, the bin mean was first computed for each participant and then averaged across participants; SE = SD of participant means/sqrt(n participants) and CI = mean ± 1.96 × SE. The number of participants contributing to each bin is shown below the x-axis. The dashed horizontal line marks the 30 breaths/min target.

### 3.2 Descriptive PetCO_2_ and ventilatory variables

PetCO_2_ fell rapidly in the first 30–60 s and then continued to decline more gradually (**Figure 3**). Participant-level mean PetCO_2_ during the maneuver was 17.1 ± 1.8 mmHg (n = 30, range 13.3–21.2 mmHg). The mean within-participant SD of PetCO_2_ was 4.5 mmHg, and the mean within-participant range was 22.3 mmHg. Descriptive statistics for PetCO_2_ and ventilatory variables are shown in **Table 2**. Mean VE was 80.7 ± 20.6 L/min, mean VCO_2_ was 1,106 ± 284 mL/min, and mean Te was 0.97 ± 0.18 s.

**Figure 3:**
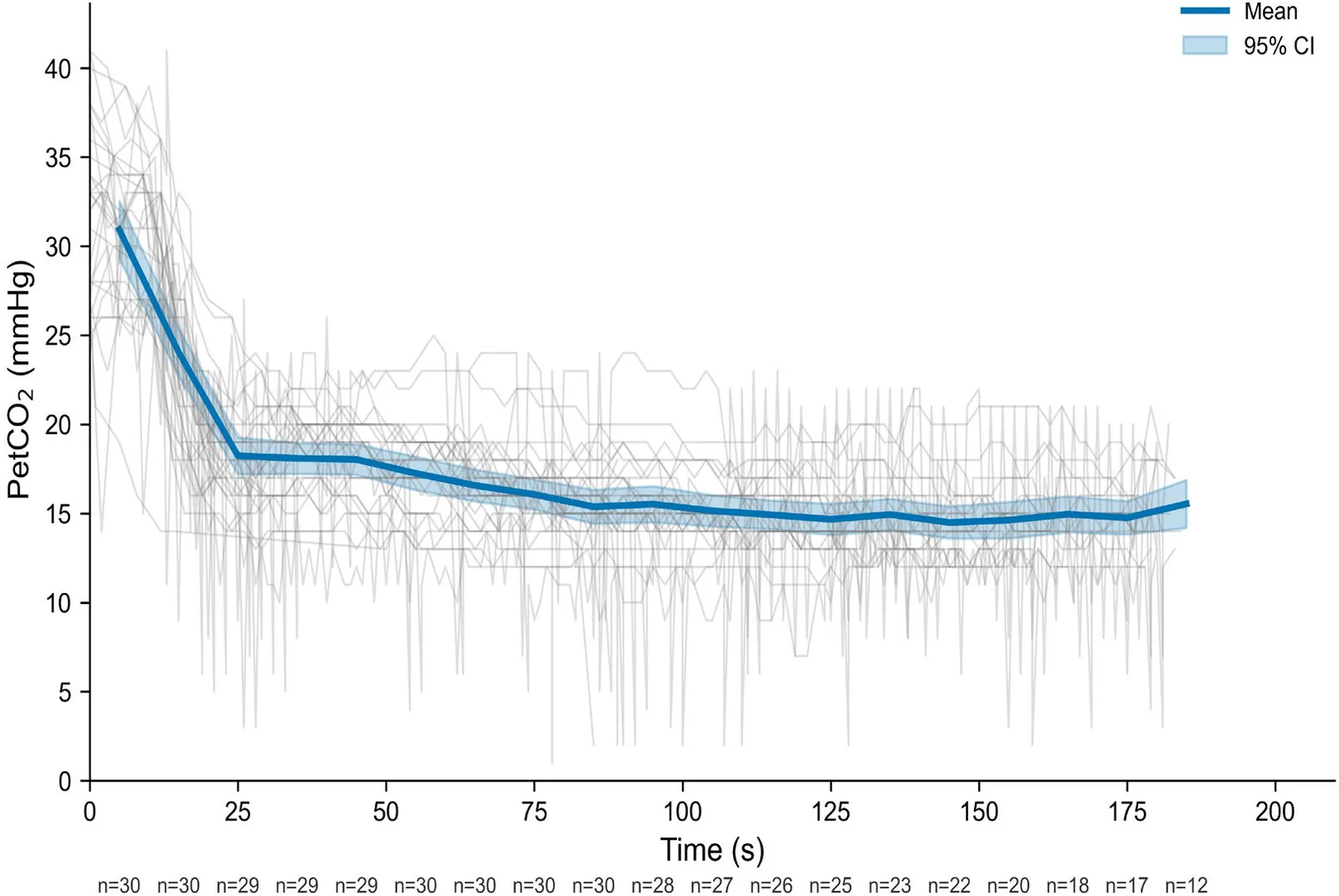
PetCO_2_ trajectories during the paced hyperventilation maneuver. Individual accepted-breath PetCO_2_ traces are shown in light gray. The solid blue line and shaded band are the mean and 95% CI within 10-s time bins; because breaths are clustered within participants, the bin mean was first computed for each participant and then averaged across participants, and the CI is ±1.96 × SE. The number of participants contributing at least one accepted breath in each bin is shown below the x-axis.

**Table 2:** Descriptive statistics of PetCO_2_ and ventilatory variables (n = 30; the mean, SD, minimum, and maximum are calculated across the 30 participant-level means).

Parameter	Mean	SD	Min	Max
PetCO_2_ (mmHg)	17.11	1.80	13.34	21.18
Respiratory frequency (breaths/min)	33.05	6.10	28.18	54.06
Tidal volume (L)	2.51	0.70	0.90	4.44
Minute ventilation (L/min)	80.73	20.64	31.33	143.37
Oxygen uptake (mL/min)	602.34	121.16	330.67	942.88
Carbon dioxide output (mL/min)	1105.65	283.78	475.73	1727.70
Expiratory time (s)	0.97	0.18	0.50	1.26
Inspiratory time (s)	0.96	0.14	0.56	1.20
Total breath duration (s)	1.94	0.24	1.19	2.24
VE/VCO_2_	73.85	11.51	56.04	115.69
VE/VO_2_	141.25	20.60	108.69	212.98

### 3.3 Repeated-measures correlations

Exploratory repeated-measures correlations, which separate within- and between-participant sources of variation, are shown in **Table 3**. Within participants, PetCO_2_ was strongly negatively correlated with elapsed time (r = −0.643, bootstrap 95% CI −0.709 to −0.578) and positively correlated with expiratory time (r = 0.617, bootstrap 95% CI 0.542 to 0.699) (**Figure 4**). VE was also negatively correlated with PetCO_2_ within participants (r = −0.518, bootstrap 95% CI −0.692 to −0.276), whereas VCO_2_ showed a weak negative within-participant correlation (r = −0.087, bootstrap 95% CI −0.265 to 0.133). Between-participant correlations differed in magnitude and sometimes direction (e.g., Te r = 0.268, bootstrap 95% CI 0.035 to 0.495; VCO_2_ r = 0.357, bootstrap 95% CI −0.077 to 0.679), underscoring the need to account for clustering and to avoid interpreting pooled associations as individual-level effects.

**Table 3:** Repeated-measures correlations of PetCO_2_ with expiratory time, ventilation, and expired CO_2_ output.

Variable X	Variable Y	N subjects	N observations	Within-participant r	95% CI (within)	Between-participant r	95% CI (between)
Te	PetCO_2_	30	2,298	0.617	(0.542, 0.699)	0.268	(0.035, 0.495)
VE	PetCO_2_	30	2,298	−0.518	(−0.692, −0.276)	−0.031	(−0.402, 0.334)
VCO_2_	PetCO_2_	30	2,298	−0.087	(−0.265, 0.133)	0.357	(−0.077, 0.679)
Elapsed time (s)	PetCO_2_	30	2,298	−0.643	(−0.709, −0.578)	0.013	(−0.276, 0.275)

Repeated-measures correlations were computed from person-mean-centered pooled data; 95% CIs were obtained by a participant-level cluster bootstrap (2,000 resamples, percentile intervals), which accounts for serial correlation within participants and unequal numbers of breaths per participant. These correlations remain exploratory because person-mean-centered pooled correlations give greater weight to participants with more breaths.

**Figure 4:**
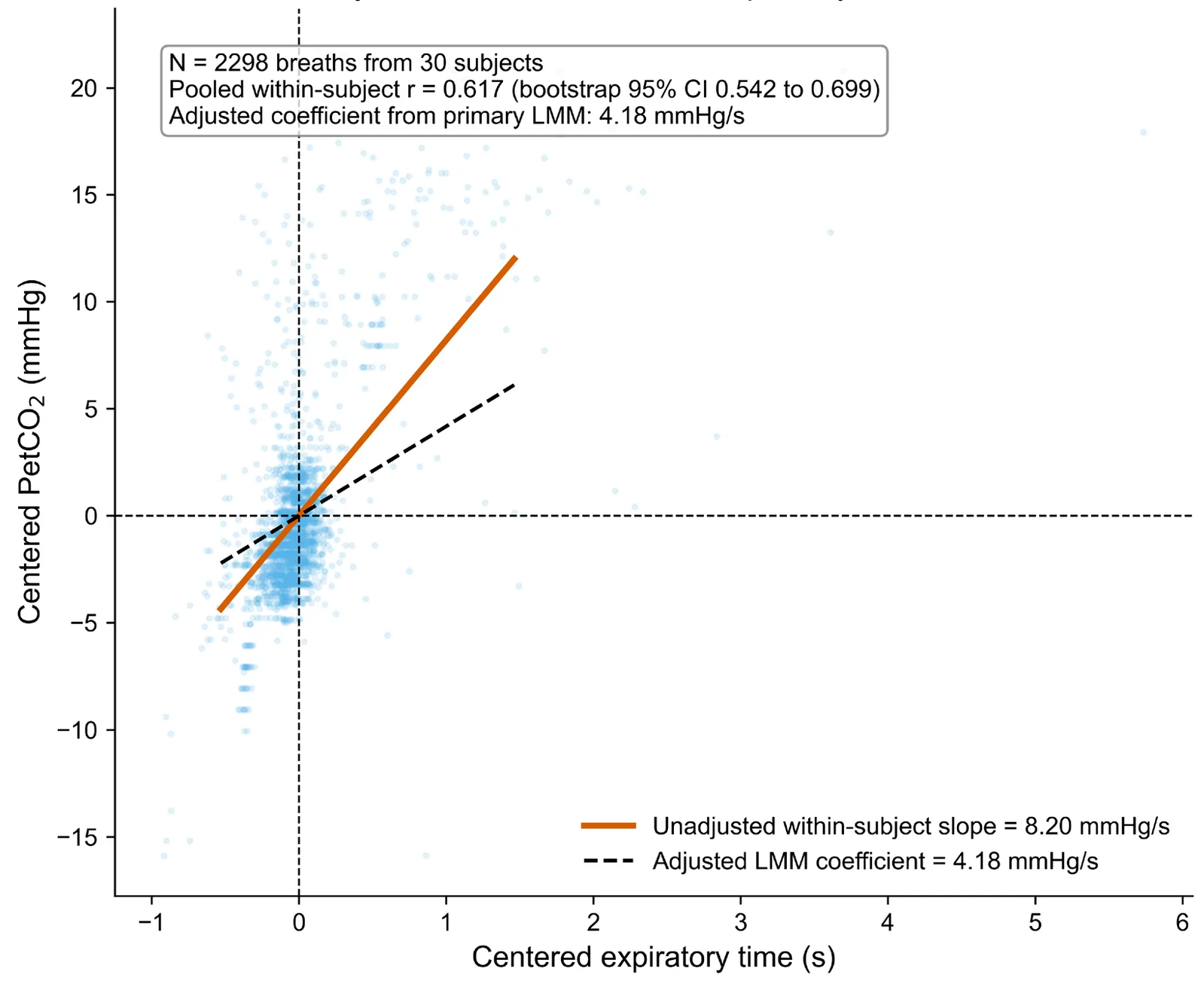
Within-participant association between expiratory time (Te) and PetCO_2_. Person-mean-centered within-participant values are shown. Each light-blue point represents one accepted breath, and each light-blue line represents one participant. The orange line is the pooled within-subject regression slope; the black dashed line shows the adjusted coefficient from the primary LMM for reference. The pooled within-subject correlation based on person-mean-centered data is descriptive and exploratory; no inferential p value is reported because breaths within a participant are serially autocorrelated.

### 3.4 Linear mixed model

Model diagnostics showed pronounced tail departures in the normal Q-Q plot and non-constant residual variance in the scale-location plot (**Figure 5**). The spline-GEE sensitivity analysis estimated an AR(1) correlation of approximately 0.79; the linear-time GEE alternative estimated an AR(1) correlation of approximately 0.86 (**Supplementary Table S2**). Consequently, cluster-bootstrap CIs are reported as the primary inferential intervals (**Table 4**). The primary LMM with a cubic B-spline for time, participant-specific random intercepts, and participant-specific random slopes for time converged and revealed fixed-effect estimates for VE, VCO_2_, and Te (**Table 4**). Wald-based 95% CIs and p-values are reported alongside cluster-bootstrap 95% confidence intervals. The spline captured a rapid early decline in PetCO_2_ followed by a plateau; the combined time effect was highly significant. The predicted change in PetCO_2_ from 0 to 180 s was −8.58 mmHg (participant-cluster bootstrap 95% CI, −10.30 to −6.92 mmHg). Higher VE was associated with lower PetCO_2_, higher VCO_2_ was associated with higher PetCO_2_, and longer Te was associated with higher PetCO_2_. The random-intercept variance was 2.23 mmHg^2^, the random-slope variance for time was 0.365 mmHg^2^/min^2^, the intercept-slope covariance was −0.662 mmHg^2^/min, the residual variance was 4.13 mmHg^2^, and the intercept-slope correlation was −0.735 (**Table 4 continued**). Because between-participant variance changes over time in a random-slope model, we report an observation-averaged VPC of 0.242 (range across observed times 0.199 to 0.350), indicating that on average 24.2% of the combined random-plus-residual variance was attributable to between-participant differences. The marginal R^2^ was 0.775 and the conditional R^2^ was 0.828, indicating that the fixed effects explained a large proportion of the variance and that adding participant-level random effects further improved fit.

**Figure 5:**
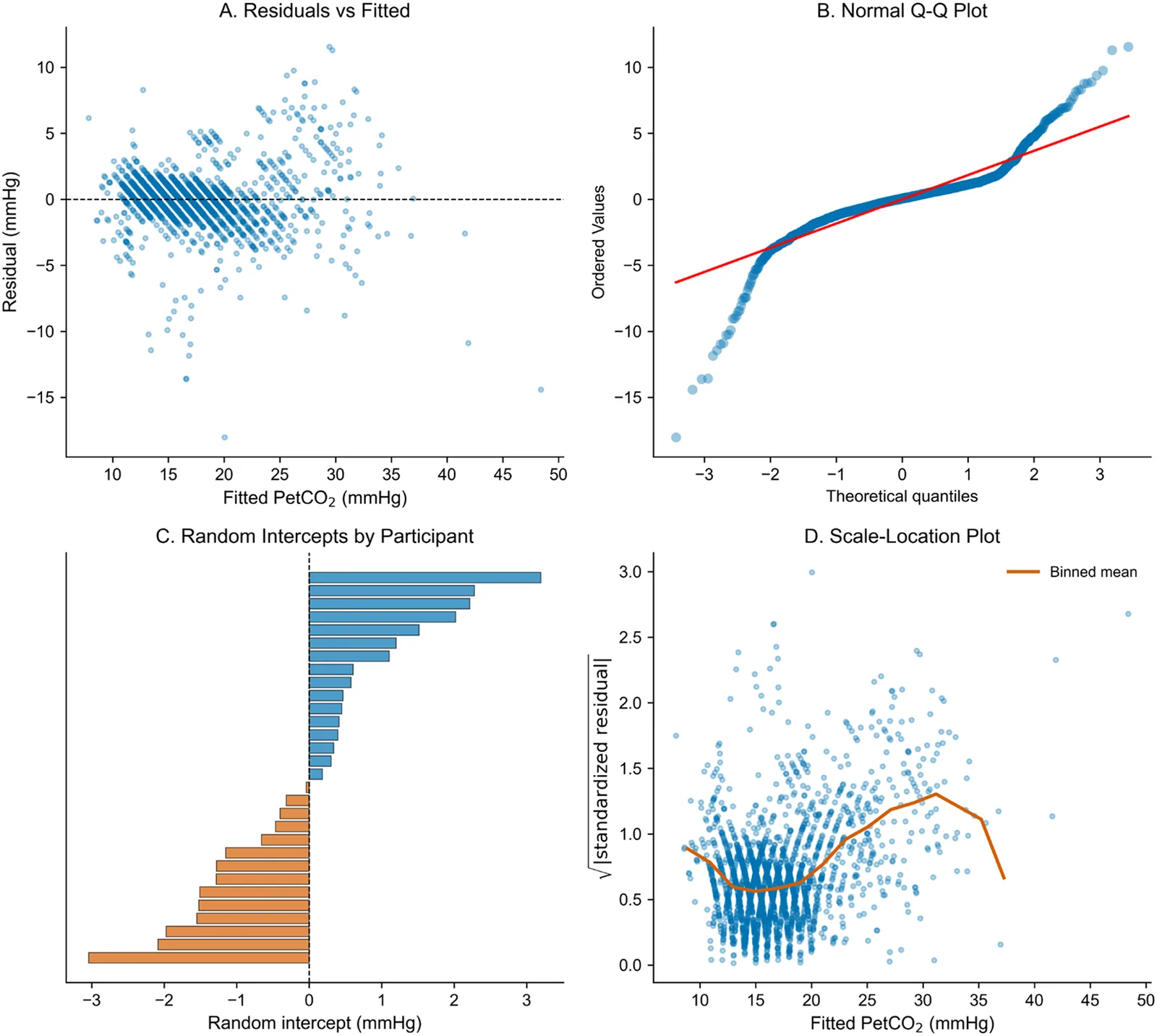
Model diagnostics for the primary LMM: **(A)** residuals versus fitted values; **(B)** normal Q-Q plot; **(C)** random intercepts by participant; **(D)** scale-location plot. Tail departures in the Q-Q plot and non-constant variance in the scale-location plot motivated the use of cluster-bootstrap CIs.

**Table 4:** CONTINUED Random-effect estimates from the primary linear mixed model predicting PetCO_2_ (n = 30 participants, 2,298 breaths).

Random effect	Estimate	Unit
Random-intercept variance	2.230	mmHg²
Random-slope variance (time)	0.365	mmHg²/min²
Intercept-slope covariance	−0.662	mmHg²/min
Intercept-slope correlation	−0.735	–
Residual variance	4.130	mmHg²

Overall nonlinear time effect: likelihood-ratio test vs. a model without time, χ²(3) = 639.71, p <0.001; vs. a model with linear time, χ²(2) = 430.14, p <0.001.

### 3.5 Within/between decomposition and sensitivity analyses

In the exploratory within/between-participant decomposition model, which used the same cubic B-spline for elapsed time as the primary model (**Table 5**), all within-participant effects were qualitatively similar to the main model, with participant-cluster bootstrap 95% CIs excluding zero for VE (β = −0.146, 95% CI −0.174 to −0.110), VCO_2_ (β = 0.009, 95% CI 0.005 to 0.012), and Te (β = 4.166, 95% CI 2.225 to 6.136). At the between-participant level, mean VE (β = −0.163, 95% CI −0.210 to −0.128) and mean VCO_2_ (β = 0.013, 95% CI 0.011 to 0.016) were associated with PetCO_2_, whereas mean Te was not (β = 1.782, 95% CI −0.471 to 4.553). This suggests that the Te–PetCO_2_ association is primarily a within-participant phenomenon rather than a between-participant difference. A spline-GEE with an AR(1) working correlation estimated an autocorrelation of approximately 0.79 and produced coefficients for VE, VCO_2_, and Te in the same direction as the primary LMM, with attenuated VE and Te magnitudes. GEE sandwich CIs were wider than LMM Wald intervals but were comparable to, not systematically wider than, the primary cluster-bootstrap intervals (**Supplementary Table S2**). A linear-time GEE was retained as an alternative model for comparison, not as a sensitivity analysis of correlation structure alone.

**Table 5:** Within/between-participant decomposition of fixed effects from the linear mixed model predicting PetCO_2_ (n = 30 participants, 2,298 accepted breaths).

Predictor	Level	Coefficient	Bootstrap 95% CI	Unit
Intercept	–	20.392	(17.883, 23.946)	mmHg
VE	Between	−0.163	(−0.210, −0.128)	mmHg per L/min
VE	Within	−0.146	(−0.174, −0.110)	mmHg per L/min
VCO_2_	Between	0.013	(0.011, 0.016)	mmHg per mL/min
VCO_2_	Within	0.009	(0.005, 0.012)	mmHg per mL/min
Te	Between	1.782	(−0.471, 4.553)	mmHg/s
Te	Within	4.166	(2.225, 6.136)	mmHg/s

Between-participant coefficients reflect associations across participant means; within-participant coefficients reflect associations of person-mean-centered deviations. Elapsed time was modeled with the same cubic B-spline basis (df = 3) as the primary model and was not decomposed; its coefficients are omitted for brevity. CIs are participant-cluster bootstrap percentile intervals (2,000 resamples). Model-based Wald SEs, CIs, and p-values are provided as supplementary comparisons. Because the coefficients have different units, they cannot be ranked by numerical magnitude.

When the analysis was restricted to the 120–180-s analysis window (603 accepted breaths from the 25 participants who reached ≥120 s), the fixed-effect estimates with participant-cluster bootstrap 95% CIs were: intercept 9.56 (6.74 to 13.98), elapsed time 0.002 (−0.007 to 0.009), VE −0.118 (−0.165 to −0.067), VCO_2_ 0.013 (0.008 to 0.016), and Te 2.072 (0.906 to 3.082) (**Supplementary Table S5**). The time coefficient was no longer significant in this late steady-state window, whereas the associations with VE, VCO_2_, and Te persisted. Two additional sensitivity analyses directly addressed adherence to the prescribed 30 breaths/min pace. Restricting the analysis to the 1,306 accepted breaths with Rf within 28–32 breaths/min (29 participants), the Te association remained positive but attenuated (β = 2.036, bootstrap 95% CI 0.917 to 3.370), with VE β = −0.187 (95% CI −0.206 to −0.170), VCO_2_ β = 0.009 (95% CI 0.008 to 0.011), and a modeled 0–180-s PetCO_2_ change of −3.97 mmHg (95% CI −7.55 to −2.73). Excluding the three participants with less than 10% of accepted breaths in the 28–32 breaths/min target range (S05, S27, S28; 27 participants, 2,099 breaths) yielded estimates essentially unchanged from the primary analysis (Te β = 4.138, 95% CI 2.137 to 6.042; modeled 0–180-s change −8.61 mmHg, 95% CI −10.54 to −6.91). The Te association therefore persists, with attenuated magnitude, under breathing patterns close to the prescribed pace. A sensitivity analysis excluding the three participants with >30% breath exclusion (S05, S26, S28; 27 participants, 2,103 accepted breaths) yielded similar coefficients (VE −0.128, VCO_2_ 0.0066, Te 2.955), indicating that the main findings were not driven by these high-exclusion participants; we note that this analysis tests sensitivity to a high exclusion proportion rather than to poor adherence to the prescribed pace. A sensitivity analysis excluding the one participant with fewer than 30 analyzable breaths yielded essentially unchanged coefficients.

### 3.6 Prediction performance

LOPO-CV of the primary LMM yielded a prediction R^2^ of 0.737 and an RMSE of 2.52 mmHg (**Figure 6**). This indicates moderate out-of-sample predictive ability, although the primary purpose of the analysis was explanatory rather than clinical prediction. The higher apparent R^2^ in the within-sample conditional model (0.828) reflects the information contributed by each participant’s own random effects, which are not available for a new participant.

**Figure 6:**
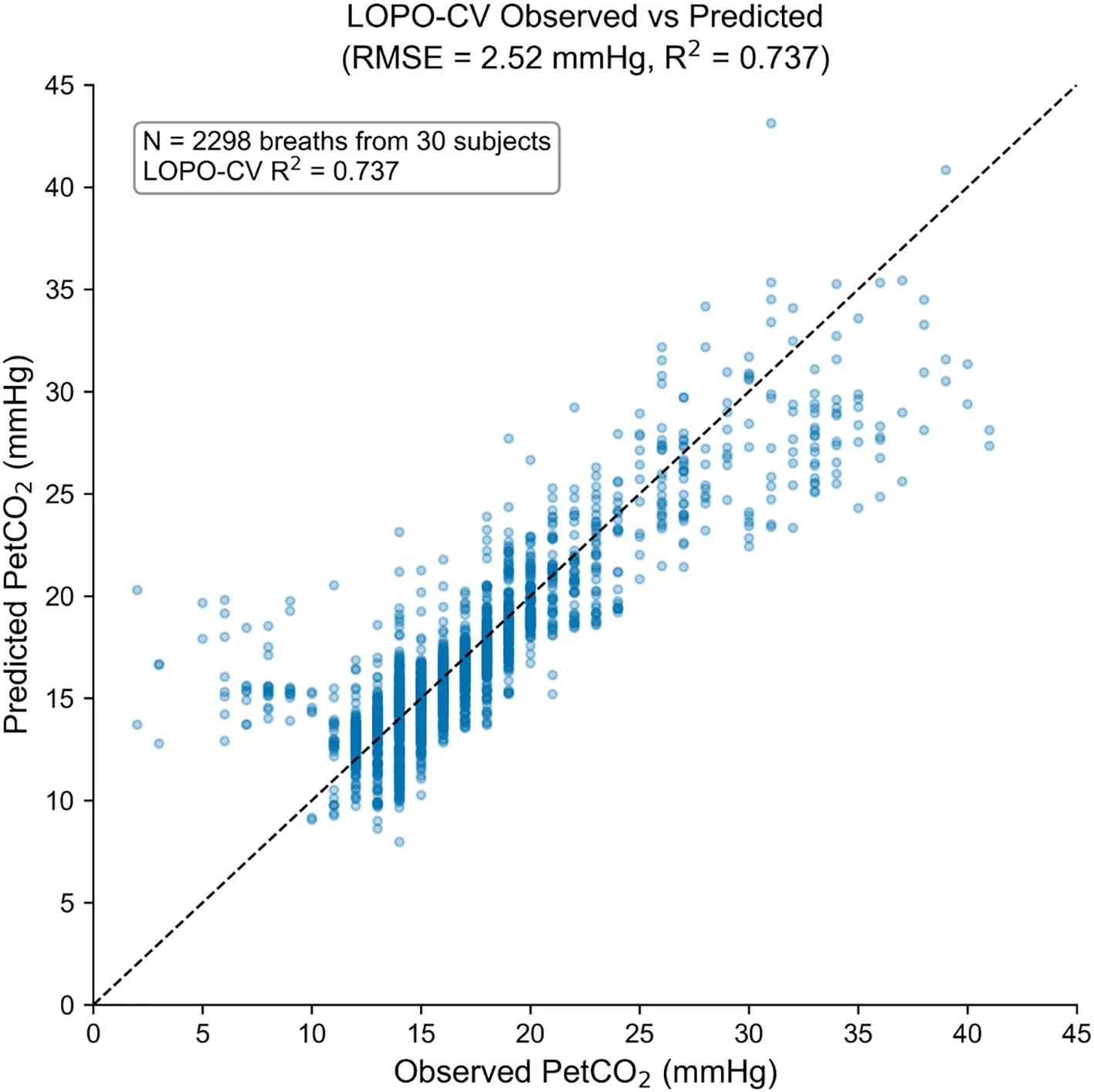
Observed versus predicted PetCO_2_ from leave-one-participant-out cross-validation of the primary LMM. Predictions are based on fixed effects only.

## 4 Discussion

This repeated-measures pilot study in 30 healthy young men showed that PetCO_2_ during paced voluntary hyperventilation was associated with elapsed time, VE, VCO_2_ output, and Te. The primary analysis used an LMM that accounted for (i) the clustering of repeated breaths within individuals, (ii) nonlinear change over time with a cubic B-spline, and (iii) participant-specific random slopes for time. The findings should be interpreted as exploratory associations and do not provide evidence of causal relationships or therapeutic mechanisms.

### 4.1 Interpretation of main associations

The negative association between VE and PetCO_2_ is consistent with the physiology of hyperventilation: greater VE removes more CO_2_ from the body stores, thereby lowering alveolar and end-tidal CO_2_ (Lum, 1975; Gardner, 1996; Brinkman et al., 2023; Yılmaz et al., 2025). The positive adjusted coefficient for VCO2 should not be interpreted straightforwardly as an inter-individual physiological effect. The unadjusted within-participant correlation between VCO_2_ and PetCO_2_ was slightly negative (r = −0.087, bootstrap 95% CI −0.265 to 0.133), whereas the adjusted within-participant coefficient was positive; this sign reversal is compatible with conditioning on correlated ventilatory variables, suppression, and measurement coupling, because VCO_2_ is calculated by the K5 as the product of expired ventilation and expired CO_2_ fraction (Brinkman et al., 2023; Sur and Hashmi, 2024). VE and VCO_2_ are algebraically coupled, and VCO_2_ and PetCO_2_ additionally share information from the expired-CO_2_ signal. The high within- and between-participant correlation between VE and VCO_2_ (median r approximately 0.85 and r approximately 0.90, respectively) means that the marginal R^2^ of the full model is partly driven by this shared measurement coupling rather than by independent physiological prediction. A model containing VE, Te, and time had a marginal R² of 0.631, a model containing VCO_2_, Te, and time had a marginal R² of 0.642, and the combined model explained 77% (**Supplementary Table S3**); therefore, the added predictive value of including both VE and VCO_2_ is modest and their coefficients should not be interpreted as independent physiological effects.

The positive association between Te and PetCO_2_ was the novel exploratory finding. A longer expiratory phase may allow more complete washout of the anatomical dead space and more reliable sampling of the alveolar plateau by the gas analyzer, thereby increasing the measured PetCO_2_ value (Bhavani-Shankar et al., 1992; Bhavani-Shankar et al., 1995; Jaffe, 1996; Restrepo et al., 2020; Altarriba et al., 2025). Alternatively, longer expiration may be associated with less overall ventilation per unit time. The within/between-participant decomposition suggests that this association is primarily within-participant, but whether it reflects a true physiological effect or a measurement artifact cannot be determined from the current data. We did not have access to continuous capnogram waveforms to confirm whether a clear phase III plateau was reached, especially during very rapid breathing.

### 4.2 Protocol adherence and implications

Only approximately half of the accepted breaths fell within the target range of 28–32 breaths/min. Even with a metronome and verbal cues, participants were unable to maintain the prescribed breathing rate. The experiment was therefore not an ideal 30 breaths/min maneuver, but rather a variety of self-modulated rapid breathing patterns. The effect of Te should therefore be interpreted cautiously because inspiratory and expiratory timing were not manipulated independently and respiratory rate and VE were not held constant. To establish a causal effect of Te on PetCO_2_ or arterial CO_2_, a future experiment would be needed in which Te is manipulated at a fixed respiratory rate and a fixed VE.

### 4.3 Limitations

This study had several limitations. First, the sample consisted of healthy young men only; women, older adults, and patients with hyperventilation syndrome or anxiety disorders were not investigated, so generalizability is limited. Second, the breath-level analyses remain observational and cannot establish causality. Third, the predictors are mathematically coupled (e.g., VE = Rf × VT, and VCO_2_ is derived from VE and expired CO_2_ fraction), so including VE and VCO_2_ together partly reflects shared measurement information rather than two independent physiological constructs. Fourth, arterial blood gases or transcutaneous CO_2_ were not measured, so we cannot confirm arterial hypocapnia or infer clinical respiratory alkalosis (Whited et al., 2023; Selki et al., 2024). Fifth, the validity of PetCO_2_ at very rapid respiratory rates depends on instrument response time, sampling-line delay, mask leak, and attainment of an alveolar plateau; these were not independently verified in the present dataset, and the original K5 export did not include continuous capnogram waveforms (Bhavani-Shankar et al., 1992; Bhavani-Shankar et al., 1995; Jaffe, 1996; Restrepo et al., 2020; Altarriba et al., 2025). Sixth, the number of independent participants (30) is small for developing a clinical prediction model, and the LOPO-CV R^2^ should be interpreted as an exploratory index rather than clinical validation. Seventh, the experiment was conducted in a controlled laboratory setting, and the findings may not translate to real-world spontaneous hyperventilation. Eighth, VT was not independently constrained; participants were instructed only to follow the metronome pace, so variation in VT contributed to both inter-individual and within-maneuver heterogeneity. Ninth, serial autocorrelation among adjacent breaths was not explicitly modeled in the primary LMM; the GEE sensitivity analyses showed high AR(1) correlations (spline-GEE alpha approximately 0.79; linear-time GEE alpha approximately 0.86), and the cluster-bootstrap CIs were wider than the Wald intervals, suggesting that model-based standard errors may be somewhat optimistic. Tenth, the number of accepted breaths per participant varied (26–97), and the temporal coverage of the 3-minute window differed across participants; a sensitivity analysis excluding the one participant with fewer than 30 accepted breaths yielded essentially unchanged coefficients. Finally, a reliable resting PetCO_2_ baseline was unavailable, because breath-by-breath recording began at the onset of the maneuver; the study therefore cannot quantify the resting-to-hyperventilation change in PetCO_2_.

### 4.4 Future directions

Future studies should include a more diverse sample, measure arterial or transcutaneous CO_2_ to validate end-tidal measurements, and collect continuous capnogram waveforms to confirm alveolar plateau quality. An interventional design that manipulates Te independently of respiratory rate and VE would be required to test whether expiratory time has a therapeutic effect on PetCO_2_. Studies in clinical populations with hyperventilation syndrome or panic disorder would be needed before any diagnostic or treatment implications can be considered (Madhusudhan et al., 2020; Leyro et al., 2021; Pauwen et al., 2022; Peiffer et al., 2022; Banushi et al., 2023; Karkouli et al., 2024; Zaliene et al., 2025).

## 5 Conclusion

In this repeated-measures pilot study of healthy young men, PetCO_2_ during paced voluntary hyperventilation was associated with elapsed time, VE, VCO_2_ output, and Te. An LMM that accounted for nonlinear time, repeated-measures clustering, and participant-level random slopes explained a large proportion of the variance. These associations are exploratory and require confirmation in larger, more diverse samples and in clinical populations before any diagnostic or therapeutic inferences can be drawn. Because VE and VCO_2_ are algebraically coupled, and VCO_2_ and PetCO_2_ also share information from the expired-CO_2_ signal, they should not be interpreted as independent physiological determinants; the principal contribution is the within-participant expiratory-time association and the description of measured PetCO_2_ dynamics.

## Data Availability

The de-identified breath-by-breath dataset and the complete Python analysis code (data processing, spline construction, random-slope VPC computation, participant-cluster bootstrap, within/between-participant decomposition, and leave-one-participant-out cross-validation) are available from the corresponding author on reasonable request. Requests to access the datasets should be directed to GuiYou Wang, 13681568909@163.com.
