# Supplementary material for: End-tidal CO2 responses and ventilatory correlates during voluntary hyperventilation: a repeated-measures pilot study
Source: Front Physiol. 2026 Sep 30;17:1890190. doi: 10.3389/fphys.2026.1890190 (PMC13661451; doi:10.3389/fphys.2026.1890190)
Supplement: Supplementary file 1 [file DataSheet1.pdf]

Supplementary Table S1

Per-participant data audit and protocol adherence. Raw breath counts, accepted breath counts, excluded breath counts, exclusion percentage, recorded duration, accepted observations per recorded minute (an analytic data-yield metric, not the participant's actual respiratory frequency), respiratory frequency calculated from consecutive accepted-breath timestamps, effective respiratory frequency (mean of K5-reported instantaneous values), percentage of accepted breaths within the 28–32 breaths/min target window, phase-specific accepted-breath counts (0–30 s, 30–120 s, 120–180 s), and mean $PetCO_2$ for the full maneuver are shown. The 120–180 s counts correspond to the common late-phase sensitivity window (603 accepted breaths from 25 participants).

Table S1. Per-participant data audit and protocol adherence (n = 30).

Subject ID	Age (years)	Height (cm)	Weight (kg)	BMI (kg/m²)	Raw breaths	Accepted breaths	Excluded breaths	Exclusion (%)	Recorded duration (s)	Accepted obs per recorded min	Rf from timestamps (breaths/min)	Effective Rf (breaths/min)	% accepted within 28–32	Breaths 0–30 s	Breaths 30–120 s	Breaths 120–180 s	Mean $PetCO_2$ (mm Hg)
S01	20.5	168.0	72.0	25.5	103	86	17	16.50	181	28.5	30.9	31.6	73.3	12	43	30	18.2
S02	34.2	182.0	70.0	21.1	87	87	0	0.00	153	34.1	38.0	34.7	26.4	14	51	22	13.3
S03	36.7	192.0	75.0	20.3	49	49	0	0.00	89	33.0	37.6	34.3	16.3	17	32	0	16.8
S04	36.9	180.0	87.5	27.0	88	88	0	0.00	180	29.3	30.0	29.4	79.5	13	44	31	19.1
S05	32.4	178.0	85.0	26.8	172	26	146	84.88	180	8.7	16.4	34.1	0.0	10	8	8	15.8
S06	20.6	173.0	78.0	26.1	90	87	3	3.33	176	29.7	30.6	30.7	77.0	15	41	31	17.2
S07	21.6	169.0	74.0	25.9	114	93	21	18.42	183	30.5	32.5	33.2	47.3	14	44	34	15.8
S08	24.5	174.0	71.0	23.5	51	48	3	5.88	102	28.2	30.7	31.2	31.2	11	37	0	16.7
S09	21.9	180.0	67.0	20.7	86	86	0	0.00	178	29.0	29.8	29.4	76.7	12	44	30	16.1
S10	20.0	173.0	74.0	24.7	98	75	23	23.47	143	31.5	33.6	33.7	18.7	15	47	13	18.3
S11	39.8	177.6	69.0	21.9	91	91	0	0.00	184	29.7	30.9	29.8	70.3	15	44	31	17.3
S12	22.5	170.0	60.0	20.8	89	88	1	1.12	181	29.2	30.8	29.5	92.0	13	44	30	17.8
S13	24.2	171.0	72.0	24.6	90	90	0	0.00	182	29.7	29.6	29.5	81.1	14	44	31	18.6
S14	19.6	168.0	66.0	23.4	67	66	1	1.49	120	33.0	35.5	33.7	42.4	16	49	1	17.1

Subject ID	Age (years)	Height (cm)	Weight (kg)	BMI (kg/m²)	Raw breaths	Accepted breaths	Excluded breaths	Exclusion (%)	Recorded duration (s)	Accepted obs per recorded min	Rf from timestamps (breaths/min)	Effective Rf (breaths/min)	% accepted within 28–32	Breaths 0–30 s	Breaths 30–120 s	Breaths 120–180 s	Mean $PetCO_2$ (mm Hg)
S15	22.7		78.0		88	88	0	0.00	179	29.5	29.6	29.7	85.2	13	45	30	16.8
S16	23.5	174.0	77.0	25.4	62	62	0	0.00	115	32.3	33.8	32.2	58.1	16	46	0	17.9
S17	23.0	172.0	77.0	26.0	47	46	1	2.13	95	29.1	32.6	29.1	60.9	15	31	0	18.6
S18	19.2	175.0	65.0	21.2	89	89	0	0.00	180	29.7	30.1	29.7	86.5	14	44	31	19.4
S19	38.8	172.0	72.0	24.3	40	40	0	0.00	85	28.2	29.5	28.2	65.0	14	26	0	17.9
S20	23.6	171.0	72.0	24.6	96	86	10	10.42	183	28.2	29.6	30.6	82.6	16	44	25	18.9
S21	27.3	175.0	71.0	23.2	48	48	0	0.00	128	22.5	31.5	30.1	79.2	8	36	4	13.4
S22	19.6	172.0	72.0	24.3	96	94	2	2.08	181	31.2	34.2	32.0	41.5	13	49	31	14.9
S23	35.9	175.0	78.0	25.5	79	79	0	0.00	159	29.8	30.6	30.5	74.7	13	45	21	14.6
S24	21.5	170.0	70.0	24.2	96	92	4	4.17	183	30.2	31.0	31.8	80.4	15	44	31	21.2
S25	39.8	169.0	80.0	28.0	74	74	0	0.00	139	31.9	37.4	34.5	20.3	10	52	12	18.3
S26	21.8	173.0	74.0	24.7	163	93	70	42.94	182	30.7	33.2	43.8	30.1	15	46	31	15.1
S27	25.2	181.0	80.0	24.4	128	97	31	24.22	147	39.6	47.4	51.2	3.1	19	64	14	17.0
S28	23.0	170.0	65.0	22.5	186	76	110	59.14	163	28.0	37.1	54.1	1.3	14	41	21	16.8
S29	25.0	173.0	78.0	26.1	86	85	1	1.16	178	28.7	29.3	29.0	68.2	11	44	30	18.9

Subject ID	Age (years)	Height (cm)	Weight (kg)	BMI (kg/m²)	Raw breaths	Accepted breaths	Excluded breaths	Exclusion (%)	Recorded duration (s)	Accepted obs per recorded min	Rf from timestamps (breaths/min)	Effective Rf (breaths/min)	% accepted within 28–32	Breaths 0–30 s	Breaths 30–120 s	Breaths 120–180 s	Mean $PetCO_2$ (mm Hg)
S30	31.4	178.0	80.0	25.2	91	89	2	2.20	179	29.8	31.1	30.2	69.7	14	45	30	15.4

Note: 'Accepted obs per recorded min' is 60 × n_accepted / recorded duration; it counts accepted breath-by-breath observations per recorded minute and is not the participant's actual respiratory frequency, because excluded observations include detected breaths or artifacts rejected on the basis of the K5-reported Rf. '% accepted within 28–32' is the percentage among accepted breaths, not among all breathing cycles during the maneuver. The phase columns count accepted breaths within 0–30 s, 30–120 s, and 120–180 s; for recordings longer than 180 s, accepted breaths occurring after 180 s are included in the total accepted count but not in any phase column (e.g., S01: 86 accepted = 12 + 43 + 30 + 1 after 180 s; S07: 93 = 14 + 44 + 34 + 1 after 180 s).

Supplementary Table S2

Generalized estimating equations were fitted with an AR(1) working correlation. The primary GEE sensitivity analysis used the same cubic B-spline time specification as the primary LMM: elapsed time was represented by a cubic B-spline basis with 3 degrees of freedom and no interior knots, generated in Patsy as bs(time_sec, df=3, degree=3); the model included a separate intercept, and the boundary knots were the minimum and maximum observed elapsed times. Observations were ordered by accepted-breath sequence within each participant, and AR(1) distance was based on this sequence; excluded breaths were removed, and irregular intervals were handled by treating consecutive accepted breaths as adjacent. The AR(1) dependence parameter (alpha) is reported in each table. The linear-time GEE is retained as an alternative model for comparison, not as a direct sensitivity analysis of correlation structure alone. Robust sandwich standard errors are reported.

Table S2a. GEE AR(1) cubic B-spline time fixed-effect estimates (n = 30 participants, 2,298 accepted breaths).

Parameter	Coefficient	SE	95% CI	P value
Intercept	23.727	1.618	(20.556 to 26.897)	<0.001
Spline basis 1	-23.737	2.148	(-27.946 to -19.527)	<0.001
Spline basis 2	-4.123	1.154	(-6.385 to -1.861)	<0.001
Spline basis 3	-13.512	1.124	(-15.714 to -11.309)	<0.001
VE	-0.097	0.016	(-0.128 to -0.066)	<0.001
VCO_2	0.0082	0.0015	(0.0052 to 0.0112)	<0.001
Te	2.525	0.620	(1.310 to 3.740)	<0.001
AR(1) alpha	0.793			

Table S2b. Linear-time GEE AR(1) fixed-effect estimates (alternative model).

Parameter	Coefficient	SE	95% CI	P value
Intercept	18.599	1.656	(15.353 to 21.845)	<0.001
time_sec	-0.050	0.004	(-0.058 to -0.041)	<0.001
VE	-0.105	0.018	(-0.139 to -0.071)	<0.001
VCO_2	0.0084	0.002	(0.005 to 0.012)	<0.001
Te	2.531	0.645	(1.267 to 3.795)	<0.001
AR(1) alpha	0.857			

Supplementary Table S3

Table S3. Model comparison for fixed-effect specification of the LMM (n = 30 participants, 2,298 breaths).

Model	AIC	BIC	Marginal R^2	Conditional R^2	VPC mean (range)
VE, Te, and time	10522.968	10580.366	0.631	0.788	0.418 (0.347–0.569)
VCO_2, Te, and time	10488.501	10545.899	0.642	0.791	0.410 (0.336–0.571)
VE, VCO_2, Te, and time	9940.200	10003.338	0.775	0.828	0.242 (0.199–0.350)
piecewise VE, VCO_2, Te, and time	10172.809	10230.207	0.756	0.817	0.268 (0.241–0.341)

The piecewise model was a bilinear model with a single knot at 60 s: $PetCO_2$ ~ time_sec + time_late + VE + VCO_2 + Te, where time_late = max(0, time_sec − 60); the slope before 60 s is the time_sec coefficient and the slope after 60 s is the sum of the two coefficients. The knot at 60 s was chosen because the descriptive trajectory showed a rapid early decline followed by a slower phase (main-text Fig. 3). The earlier VE + $FECO_2$ + Te + time model was removed: $FECO_2$ derived as VCO_2/VE is not directly interpretable as a fractional expired CO_2 concentration because ventilatory volumes are reported at BTPS whereas gas-exchange variables are reported at STPD, and the resulting design matrix contained missing or rank-deficient columns.

Supplementary Table S4

Table S4. Within/between-participant decomposition LMM with cubic B-spline time (main-text Table 5): model-based Wald results, shown as supplementary comparisons. The primary inferential intervals for this model are the participant-cluster bootstrap CIs in Table 5. Fixed-effects specification: $PetCO_2$ ~ bs(time_sec, df=3, degree=3) + VE_m + VE_cw + VCO_2_m + VCO_2_cw + Te_m + Te_cw; random intercept and random slope for time (time_min); fitted by maximum likelihood.

Parameter	Coefficient	SE	95% CI (Wald)	P value (Wald)
Intercept	20.392	1.0278	(18.377, 22.406)	<0.001
bs(time_sec, df=3, degree=3)[0]	-14.327	0.6540	(-15.609, -13.046)	<0.001
bs(time_sec, df=3, degree=3)[1]	-1.725	0.4548	(-2.616, -0.833)	<0.001
bs(time_sec, df=3, degree=3)[2]	-8.928	0.5323	(-9.971, -7.884)	<0.001
VE_m	-0.163	0.0190	(-0.200, -0.125)	<0.001
VE_cw	-0.146	0.0060	(-0.158, -0.134)	<0.001
VCO_2_m	0.013	0.0014	(0.010, 0.016)	<0.001
VCO_2_cw	0.009	0.0004	(0.008, 0.010)	<0.001
Te_m	1.782	0.9356	(-0.051, 3.616)	0.057
Te_cw	4.166	0.1416	(3.888, 4.443)	<0.001

Supplementary Table S5

Table S5. Late-window (120–180 s) LMM (random intercept and random slope for time; 603 accepted breaths from the 25 participants who reached ≥120 s): model-based Wald and participant-cluster bootstrap 95% CIs (2,000 resamples, percentile intervals; duplicate draws received new unique cluster identifiers; all 2,000 fits converged). The bootstrap intervals are the primary inferential intervals.

Parameter	Coefficient	95% CI (Wald)	P value (Wald)	Bootstrap 95% CI
Intercept	9.557	(8.150, 10.964)	<0.001	(6.739, 13.977)
time_sec	0.002	(-0.005, 0.010)	0.565	(-0.007, 0.009)
VE	-0.118	(-0.135, -0.102)	<0.001	(-0.165, -0.067)
VCO_2	0.013	(0.011, 0.014)	<0.001	(0.008, 0.016)
Te	2.072	(1.621, 2.524)	<0.001	(0.906, 3.082)

Supplementary Table S6

Table S6. Protocol-adherence sensitivity analyses. Both analyses used the same cubic B-spline time specification and random-effects structure as the primary LMM. CIs are participant-cluster bootstrap percentile intervals (2,000 resamples). 'time_effect' is the modelled change in $PetCO_2$ from 0 to 180 s with other predictors held at zero.

Analysis / Parameter	Coefficient	Bootstrap 95% CI
(a) Only accepted breaths with Rf 28–32 /min (29 participants, 1,306 breaths)		
VE	-0.187	(-0.206, -0.170)
VCO_2	0.009	(0.008, 0.011)
Te	2.036	(0.917, 3.370)
time_effect	-3.972	(-7.547, -2.734)
(b) Excluding S05, S27, S28 (<10% of accepted breaths in 28–32 /min; 27 participants, 2,099 breaths)		
VE	-0.139	(-0.170, -0.101)
VCO_2	0.009	(0.005, 0.012)
Te	4.138	(2.137, 6.042)
time_effect	-8.614	(-10.540, -6.911)

Supplementary data and code availability. The de-identified breath-by-breath dataset and the complete Python analysis code (data processing, spline construction, random-slope VPC computation, participant-cluster bootstrap, within/between-participant decomposition and leave-one-participant-out cross-validation) are available from the corresponding author on reasonable request.
